# Isotopic and genetic evidence of partial mycoheterotrophy in leafy variants of *Cremastra aphylla*, a predominantly leafless orchid

**DOI:** 10.1093/aob/mcaf235

**Published:** 2025-09-25

**Authors:** Kenji Suetsugu, Hidehito Okada, Shun K Hirota, Yoshihisa Suyama

**Affiliations:** Department of Biology, Graduate School of Science, Kobe University, Kobe 657-8501, Japan; Institute for Advanced Research, Kobe University, Kobe 657-8501, Japan; Department of Biology, Graduate School of Science, Kobe University, Kobe 657-8501, Japan; Botanical Gardens, Osaka Metropolitan University, Osaka 576-0004, Japan; Graduate School of Symbiotic Systems Science and Technology, Fukushima University, Fukushima 960-1296, Japan; Field Science Center, Graduate School of Agricultural Science, Tohoku University, Miyagi 989-6711, Japan

**Keywords:** Chlorophyll fluorescence, mixotrophy, mycoheterotrophy, Orchidaceae, partial mycoheterotrophy, Psathyrellaceae, stable isotopes, trophic plasticity

## Abstract

**Background and Aims:**

The evolution of mycoheterotrophy represents a major evolutionary transition that is likely to proceed through intermediate stages, such as initial and partial mycoheterotrophy. *Cremastra aphylla*, a predominantly leafless, fully mycoheterotrophic orchid, occasionally produces individuals with well-developed green leaves that are likely to contain chlorophyll. However, it remains unclear whether these forms represent phenotypic variants or an undescribed taxon distinct from true *C. aphylla*. Additionally, given the occasional co-occurrence of *C. aphylla* with the closely related, leafy *Cremastra appendiculata*, these individuals might have regained some photosynthetic capacity via hybridization or introgression.

**Methods:**

We integrated chlorophyll concentration and fluorescence measurements, stable isotope (δ^13^C and δ^15^N) analysis, MIG-seq-based phylogenomics and metabarcoding of mycorrhizal partners to investigate the physiological ecology and evolutionary background of both leafless and leafy *C. aphylla* individuals.

**Key Results:**

Both morphotypes exhibited elevated δ^13^C values in comparison to co-occurring autotrophic species. However, leafy individuals showed significantly lower values, indicating the presence of ^13^C-depleted photosynthates. A two-source mixing model based on ^13^C enrichment indicated that photosynthesis contributed ∼40 % of leaf carbon and ∼20 % of floral carbon. Chlorophyll analyses confirmed active chlorophyll synthesis and photosystem II efficiency in leafy individuals, with values comparable to those of autotrophic plants. Fungal metabarcoding revealed that both morphotypes, each bearing coralloid rhizomes, were associated with the same Psathyrellaceae operational taxonomic unit, probably *Coprinellus magnoliae*. MIG-seq analysis detected no introgression with *C. appendiculata* and no genetic differentiation between the morphotypes, ruling out the possibility that the leafy form represents either an introgressed lineage or an undescribed taxon.

**Conclusions:**

These findings provide the first integrated isotopic and genetic evidence for partial mycoheterotrophy in leafy *C. aphylla* individuals, although they remain highly dependent on fungal carbon. Our results refine the current understanding of the nutritional continuum in partially mycoheterotrophic orchids and highlight *C. aphylla* as a valuable model for investigating the evolutionary transition towards full mycoheterotrophy.

## INTRODUCTION

The evolution of mycoheterotrophic plants that have entirely lost photosynthetic capacity is one of the most intriguing topics in plant evolution (Merckx, 2013). This trait is especially common in Orchidaceae, where >1 % of species have fully abandoned photosynthesis (Merckx, 2013). Orchid seeds lack endosperm and depend entirely on fungal partners for nutrients during early development, a condition termed initial mycoheterotrophy (Merckx, 2013; Dearnaley *et al.*, 2016). This dependence predisposes orchids to evolve life-long mycoheterotrophy (Leake, 1994). Notably, many chlorophyllous orchids also acquire carbon from mycorrhizal fungi in adulthood, a strategy known as partial mycoheterotrophy (Gebauer and Meyer, 2003; Bidartondo *et al.*, 2004; Julou *et al.*, 2005).

The transition from autotrophy to full mycoheterotrophy is likely to proceed gradually, involving intermediate stages of varying degrees (Jacquemyn and Merckx, 2019). This complex evolutionary process entails coordinated shifts across multiple phenotypic traits. Characteristics associated with full mycoheterotrophy include shifts in mycorrhizal partners (Bidartondo *et al.*, 2004), reduced vegetative structures such as leaves and roots (Imhof *et al.*, 2013; Tsukaya, 2018), altered dormancy patterns (Roy *et al.*, 2013; Shefferson *et al.*, 2024), enhanced pathogen and herbivore defences (Klooster *et al.*, 2009; Roy *et al.*, 2013), modified reproductive strategies (Waterman *et al.*, 2013; Suetsugu, 2022) and simplified seed structures, including loss of cotyledons and endosperm (Eriksson and Kainulainen, 2011). Some orchids, particularly those in shaded understoreys, acquire carbon from ectomycorrhizal networks or from wood- and litter-decaying fungi, which probably enhances fitness and promotes gradual reductions in photosynthetic investment (Bidartondo *et al.*, 2004; Suetsugu *et al.*, 2022; Suetsugu and Okada, 2025*a*).

Stable isotope analysis has become a key tool for detecting partial mycoheterotrophy, because fungal symbionts are typically enriched in heavy isotopes, such as ^2^H, ^13^C and ^15^N, owing to preferential incorporation of heavy isotopes into fungal tissues during metabolism, elevating their isotopic signatures relative to surrounding plants (Gebauer and Meyer, 2003; Gebauer *et al.*, 2016; Gomes *et al.*, 2023; Zahn *et al.*, 2023). Fully mycoheterotrophic plants show isotope values closely matching those of their fungal partners, and partially mycoheterotrophic species exhibit values intermediate between autotrophs and full mycoheterotrophs (Gebauer and Meyer, 2003; Bidartondo *et al.*, 2004; Julou *et al.*, 2005). Recent studies indicate that partial mycoheterotrophy spans a continuum from predominantly autotrophic to almost fully heterotrophic nutrition (Gebauer *et al*., 2016; Jacquemyn and Merckx, 2019). This variation is often correlated with leaf development, ranging from fully formed leaves to highly reduced structures; however, leaf morphology alone is not a reliable predictor of trophic mode, because some orchids with large leaves are strongly dependent on fungal carbon (Selosse *et al.*, 2004; Sakamoto *et al.*, 2016; Suetsugu *et al.*, 2021*a*, 2022). The transition to full mycoheterotrophy is likely to have occurred through gradual evolutionary steps rather than a single genetic change (Roy *et al.*, 2013; Suetsugu *et al.*, 2018).

Certain orchids, such as *Limodorum abortivum*, *Cymbidium macrorhizon* and *Corallorhiza trifida*, lack foliage leaves and retain only scale leaves (hereafter referred to as leafless, following orchid terminology; Ogura-Tsujita *et al.*, 2021), yet they possess green stems and capsules with residual photosynthetic capacity (Girlanda *et al.*, 2006; Zimmer *et al.*, 2008; Suetsugu *et al.*, 2018; Kobayashi *et al.*, 2021; Suetsugu *et al*., 2024). For example, *Cymbidium macrorhizon* derives ∼25 % of its carbon through photosynthesis during fruiting (Suetsugu *et al.*, 2018). These chlorophyll-retaining but leafless species might represent advanced stages in the transition towards full mycoheterotrophy.

It is also noteworthy that partially mycoheterotrophic orchids occasionally produce albino individuals or variegated individuals (with leaves showing mosaic chlorophyll loss) that are comparable in size to their green counterparts (Julou *et al.*, 2005; Selosse and Roy, 2009; Stöckel *et al.*, 2011). Although these variants exhibit reduced fitness, they offer valuable models for investigating physiological transitions towards full heterotrophy within a shared genetic framework (Roy *et al.*, 2013; Suetsugu *et al.*, 2017; Lallemand *et al.*, 2019*b*). A contrasting phenomenon involves the emergence of leafy individuals in species generally regarded as leafless and fully mycoheterotrophic. For instance, although typically *Pyrola aphylla* (Ericaceae) is fully mycoheterotrophic and reliant on ectomycorrhizal fungi, some individuals display phenotypic plasticity by producing small green leaves (Zimmer *et al.*, 2007; Hynson *et al.*, 2009). Although δ^13^C isotopic evidence suggests that these leaves contribute minimally to carbon acquisition, this conclusion is based on only two individuals (*n* = 2; Hynson *et al.*, 2009) and requires further investigation. Moreover, whether similar patterns occur in distantly related fully mycoheterotrophic lineages, such as orchids, remains unexplored.

This study focuses on *Cremastra aphylla*, a predominantly leafless, fully mycoheterotrophic species that associates with wood-decaying Psathyrellaceae fungi (Yagame *et al.*, 2018; Suetsugu *et al.*, 2022). Its classification as fully mycoheterotrophic is supported by the absence of normal leaves, dark violet pigmentation and pronounced ^13^C enrichment, exceeding that of most known fully mycoheterotrophic species (Merckx, 2013; Yagame *et al.*, 2018; Ogura-Tsujita *et al.*, 2021; Suetsugu *et al.*, 2022). The *Cremastra appendiculata* species complex, comprising *C. appendiculata* (var. *appendiculata* and var. *variabilis*) and *C. aphylla*, serves as a useful model for studying the evolution of full mycoheterotrophy. This is attributable to two factors: (1) their sister-species relationship, with some authors even treating *C. aphylla* as an intraspecific variant of *C. appendiculata* despite differences in floral morphology; and (2) the ability of the typically autotrophic *C. appendiculata* to shift towards strong mycoheterotrophy when associated with Psathyrellaceae fungi, which also form symbioses with *C. aphylla* (Inoue, 1957; Yagame *et al.*, 2021; Zahn *et al.*, 2022; Suetsugu *et al.*, 2022; Suetsugu and Okada, 2025*b*). In *C. appendiculata*, the degree of mycoheterotrophy varies with subterranean morphology and fungal partners: individuals with coralloid rhizomes often associate with Psathyrellaceae and are partially mycoheterotrophic, whereas those with only roots typically associate with rhizoctonias and are fully autotrophic even in hydrogen isotope analyses (Zahn *et al.*, 2022). This variation illustrates the nutritional continuum within the species complex.

An intriguing but understudied phenomenon in this species complex is the rare occurrence (<20 %) of *C. aphylla* individuals with well-developed green leaves (hereafter referred to as leafy *C. aphylla*) (Inoue, 2016). The extent to which these individuals rely on photosynthesis and whether their fungal associations differ from those of leafless individuals remains unknown. It is also unclear whether they represent phenotypic variants or a distinct taxonomic entity. One possibility is that leafy forms correspond to an undescribed taxon with an independent evolutionary origin (Kobayashi *et al.*, 2021). Additionally, given that *C. aphylla* occasionally co-occurs with the closely related, large-leaved *C. appendiculata*, they might have arisen through hybridization or introgression with that taxon (e.g. Ogura-Tsujita *et al.*, 2014). Alternatively, as in *Pyrola aphylla*, these leafy individuals might simply reflect phenotypic plasticity within *C. aphylla*.

This study integrates chlorophyll measurements, stable isotope analyses (δ^13^C and δ^15^N), MIG-seq (multiplexed ISSR genotyping by sequencing)-based phylogenetics and fungal metabarcoding to investigate the ecophysiology and evolutionary background of leafy *C. aphylla*. Specifically, MIG-seq was used to assess genetic differentiation and potential hybridization among leafy *C. aphylla*, leafless *C. aphylla* and *C. appendiculata*. The trophic status of leafy *C. aphylla* was evaluated using chlorophyll content, fluorescence and δ^13^C and δ^15^N isotopic signatures. Lastly, fungal associates of both leafy and leafless individuals were identified through high-throughput DNA sequencing.

## MATERIALS AND METHODS

### Study species

*Cremastra aphylla* is a fully mycoheterotrophic orchid endemic to the cool temperate forests of Hokkaido, Honshu and Shikoku, Japan (Inoue, 2016). It typically lacks foliage and is generally classified as a leafless orchid (Merckx, 2013; Yagame *et al.*, 2018; Ogura-Tsujita *et al.*, 2021; Suetsugu *et al.*, 2022). It occasionally co-occurs with the closely related *C. appendiculata*, which produces a single 20–35 cm green leaf outside the flowering season (Inoue, 2016; Suetsugu *et al.*, 2022). *Cremastra appendiculata* can be divided into two varieties (*C. appendiculata* var. *appendiculata* and *C. appendiculata* var. *variabilis*) with all Japanese individuals classified as var. *variabilis* (Lund, 1988; Inoue, 2016). The labellum of *C. aphylla* bears a stout, rugose–verrucose callus ∼5 mm in length, whereas that of *C. appendiculata* var. *variabilis* features a slender, smooth callus measuring 2–4 mm (Inoue, 2016). Additionally, the column of *C. aphylla* lacks the ventral wings present in *C. appendiculata* var. *variabilis* (hereafter *C. appendiculata*) (Yukawa, 1999; Inoue, 2016; Suetsugu, 2021).

**Fig. 1.**
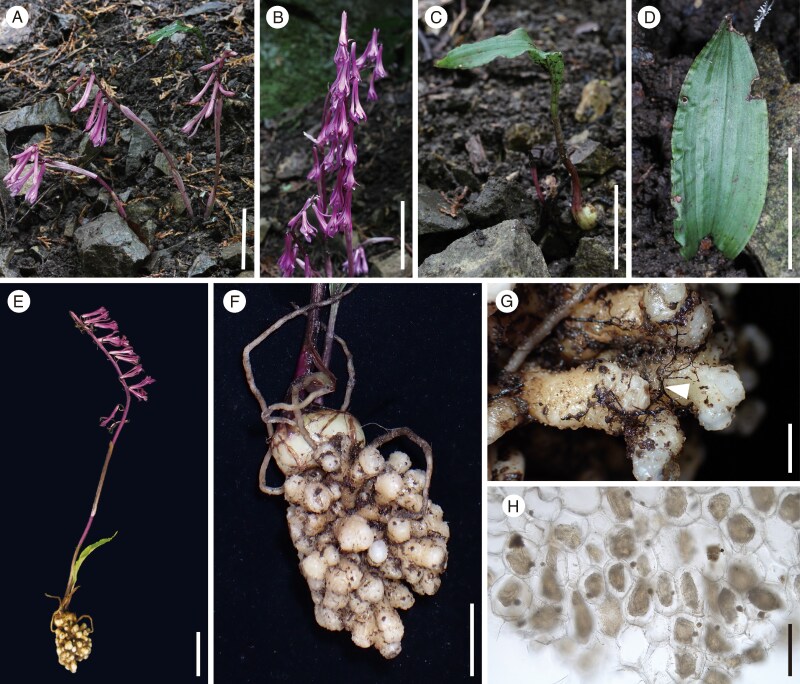
*Cremastra aphylla* and its mycorrhizal interaction. (A) Leafless individuals. (B) Close-up of the inflorescence in a leafless individual. (C, D) Leafy non-flowering individuals. (E) Leafy flowering individual. (F) Close-up of underground parts, including the corm, roots, and coralloid rhizome. (G) Close-up of a coralloid rhizome. Arrows indicate rhizomorphs of a *C. aphylla* mycorrhizal fungus. (H) Cross-section of coralloid rhizome showing degenerated fungal coils. Scale bars: 5 cm in A–E; 2 cm in F; 5 mm in G; and 200 μm in H.

The taxonomic identity of *C. aphylla* remains somewhat contentious. Some authors restrict *C. aphylla* to individuals lacking fully developed leaves, treating leafy forms as a potentially undescribed taxon (Kobayashi *et al.*, 2009). Others include individuals with small leaves (∼10 cm in length) within *C. aphylla* if they exhibit key morphological traits, such as dark purple flowers, a stout rugose–verrucose callus on the labellum and the absence of ventral wings on the column (Inoue, 2016). Some researchers have proposed that *C. aphylla*, including leafless individuals, should be regarded as an intraspecific variant of *C. appendiculata* rather than as a distinct species (Inoue, 1957). Following Inoue (2016), we identified individuals bearing small leaves, dark purple flowers and a stout rugose–verrucose callus on the labellum and lacking ventral wings on the column as *C. aphylla* (Fig. 1).

### Field study

Fieldwork, including chlorophyll measurements, in addition to sampling for stable isotope (δ^13^C and δ^15^N) analysis and mycorrhizal metabarcoding, was conducted on 22 May 2021 in a *C. aphylla* population in Shimoongatamachi, Hachioji City, Tokyo Prefecture, Japan (Fig. 1). The population included ∼30 leafless flowering, 5 leafy flowering and 5 leafy non-flowering individuals (Fig. 1). To minimize disturbance during rhizome inspection, ∼20 cm of soil was carefully excavated laterally around selected individuals. All examined plants (ten leafless flowering, four leafy flowering and four leafy non-flowering) consistently possessed coralloid rhizomes (highly branched subterranean stems), which serve as the primary sites of mycorrhizal formation in *Cremastra* (Suetsugu *et al.*, 2022). Because these rhizomes contain abundant mycorrhizal pelotons, they were used for molecular barcoding of fungal partners.

For stable isotope analysis, four 2 m × 2 m quadrats were established around both leafless and leafy individuals. This quadrat size was selected to reduce microhabitat heterogeneity while allowing comprehensive sampling of reference plants. Following Gebauer and Meyer (2003), leaf samples from representative understorey species at a height similar to that of the focal species were collected to establish reliable baseline isotope values. In total, 52 specimens were collected: 10 flowers and 5 rhizomes from leafless flowering *C. aphylla*; 5 flowers, 3 leaves and 3 rhizomes from leafy flowering individuals; 3 leaves from leafy non-flowering individuals; and 23 autotrophic reference plants.

Chlorophyll content and fluorescence in leaves of *C. aphylla* and co-occurring autotrophic species (*Aucuba japonica* var. *japonica*, *Orixa japonica* and *Rubia argyi*; *n* = 6 each) were measured using a SPAD-502 chlorophyll meter (Konica Minolta Sensing Inc., Osaka, Japan) and a FluorPen FP100 fluorometer (Photon Systems Instruments, Brno, Czech Republic), following Shutoh *et al*. (2020) and Suetsugu *et al.* (2021*b*). After confirming no major deviations from normality and homoscedasticity with Q–Q plots and DHARMa simulation-based diagnostics, we tested differences in chlorophyll metrics between *C. aphylla* and autotrophic species using ANOVA with Tukey–Kramer *post hoc* tests for pairwise comparisons. Each response variable was analysed separately. All statistical analyses were performed in R (R Core Team, 2025).

**Fig. 2.**
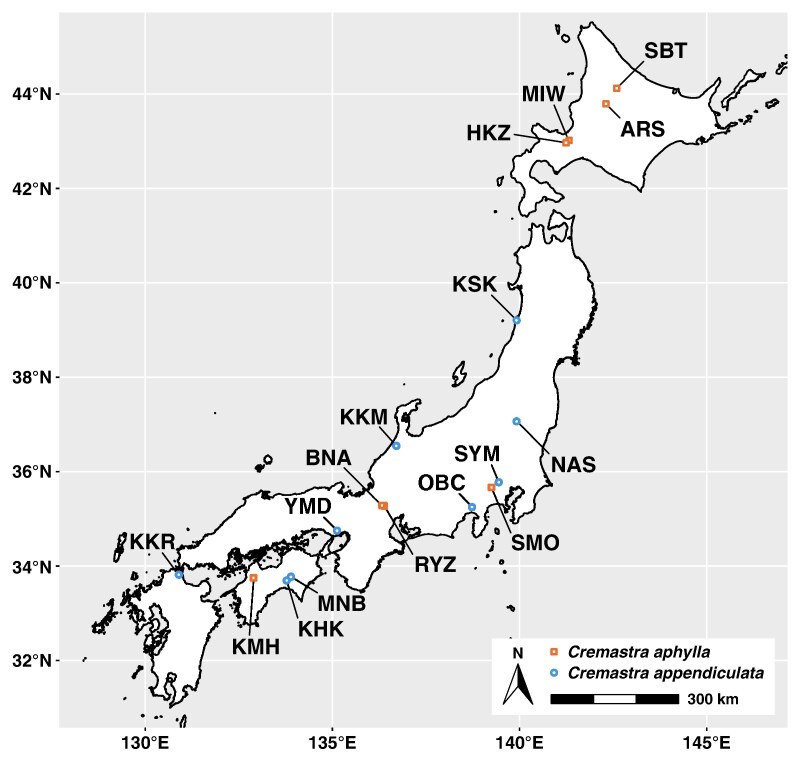
Map showing the sampling localities of *Cremastra aphylla* and its close relative *Cremastra appendiculata*. Population ID details are provided in Table S1.

Moreover, owing to the taxonomic ambiguities surrounding leafy *C. aphylla* individuals, we sampled *C. aphylla* and *C. appendiculata* across Japan from June 2010 to May 2021 (Fig. 2) to clarify the phylogenetic identity of our focal specimens [leafy *C. aphylla* from the Shimoongatamachi population (SMO)] using MIG-seq analysis (see below). Voucher specimens (at least one per population) were deposited in the herbarium of the Kyoto University Museum (KYO), Japan (Supplementary Data Table S1).

### Phylogenomic analysis

To clarify the phylogenetic identity of leafy *C. aphylla*, we conducted MIG-seq, a reduced-representation sequencing method. A library was constructed for 68 *Cremastra* samples, including four leafy *C. aphylla* individuals from one population, 26 leafless individuals from eight populations, and 38 *C. appendiculata* individuals from nine populations (Supplementary Data Table S1), following Suyama *et al.* (2022). Sequencing was performed on the Illumina MiSeq platform using the MiSeq Reagent Kit v.3 (2 × 75 bp), and raw data were submitted to the DDBJ Sequence Read Archive (accession number PRJDB35934).

After adapter and primer trimming and stringent quality filtering, we retained 13 958 316 high-quality reads (mean ± s.d.: 205 269 ± 6310 per sample) from 15 343 430 total reads (225 639 ± 7304). Single-nucleotide polymorphism (SNP) calling was performed *de novo* using the Stacks v.2.68 pipeline (Rochette *et al.*, 2019), with the following parameters: minimum stack depth (*m*) = 3; maximum stack distance (*M*) = 2; and catalogue mismatch allowance (*n*) = 2. To improve data quality, we excluded SNPs with high observed heterozygosity (*H*_o_ ≥ 0.6) and those with a minor allele count of fewer than three, retaining only SNPs present in ≥34 samples (loci genotyped in ≥50 % of individuals) using the populations module. To reduce linkage disequilibrium, we applied PLINK v.1.90 (Chang *et al.*, 2015) with the option –indep-pairwise 50 10 0.1. In total, 1513 SNPs from 68 individuals were used for subsequent analysis. Additionally, to investigate the genetic structure within *C. aphylla*, SNP detection was performed separately for 30 individuals of *C. aphylla*. After excluding SNPs missing in >15 individuals, 259 SNPs were retained.

To assess genetic differentiation, we conducted SNP-based analyses including maximum-likelihood phylogeny, Neighbor-Net network construction and STRUCTURE-based clustering using two datasets: the full sample set and the *C. aphylla*-only subset. Maximum-likelihood phylogenetic analysis was performed using RAxML v.8.2.10 (Stamatakis, 2014) under the GTR model with Lewis correction for ascertainment bias and 1000 bootstrap replicates. A Neighbor-Net network was generated in SplitsTree App v.6.4.13 (Huson and Bryant, 2006) using an uncorrected *p*-distance matrix with ambiguous sites excluded.

Population structure was inferred using STRUCTURE v.2.3.4 (Pritchard *et al.*, 2000), using an admixture model with 30 replicates per genetic cluster (*K*) value. Each run included a burn-in of 100 000 steps and 100 000 Markov chain Monte Carlo iterations. The optimal number of clusters was determined using the Δ*K* method (Evanno *et al.*, 2005) via STRUCTURE HARVESTER (Earl and vonHoldt, 2012), and results were visualized with CLUMPAK (Kopelman *et al.*, 2015).

### Molecular identification of mycorrhizal fungi

Mycorrhizal fragments containing fungal pelotons (3–5 mm in length) were collected from each *C. aphylla* sample (*n* = 8 for leafless individuals and *n* = 6 for leafy individuals) for molecular analysis. The fragments were surface-sterilized, and DNA was extracted using the CTAB method (Doyle and Doyle, 1990).

We followed the protocol described by Suetsugu and Okada (2025*a*) to amplify the ITS region of fungal DNA. The first PCR used the ITS86F/ITS4 primer set, each fused with 3–6-mer random nucleotides and Illumina sequencing primers. A second PCR was then conducted to attach Illumina P5/P7 adapters and unique sample indices. The resulting amplicon library was sequenced on an Illumina MiSeq platform using the MiSeq Reagent Micro Kit v.2 (300 cycles). The raw sequence reads have been deposited in the NCBI Sequence Read Archive (accession no. PRJNA1213263).

Bioinformatic analyses were performed using Claident v.0.9.2024.06.10 (Tanabe and Toju, 2013), following the approach of Suetsugu and Okada (2025*a*). Briefly, high-quality reads were clustered into operational taxonomic units (OTUs) using a 97 % similarity threshold. Taxonomic assignments were made to the genus level when possible, and OTUs identified as orchid mycorrhizal fungi (Dearnaley *et al.*, 2012; Wang *et al.*, 2021) were retained for further analysis. A maximum-likelihood tree of the dominant OTU and its closest relatives was constructed using IQ-TREE v.2.2.2, with the best-fitting model selected via ModelFinder (Kalyaanamoorthy *et al.*, 2017). Node support was assessed using SH-aLRT and ultrafast bootstrap (UFboot) methods.

### δ^13^C and δ^15^N analysis

The natural abundances of ^13^C and ^15^N in *C. aphylla* and its neighbouring autotrophic plants were measured using a continuous-flow isotope-ratio mass spectrometer connected to an elemental analyser (Thermo Fisher Scientific, Waltham, MA, USA), following Suetsugu and Matsubayashi (2021). Relative isotope abundances were calculated as:


$$\delta^{13}C\;\mathrm{or}\;\delta^{15}N=(R_{\mathrm{sample}}/R_{\mathrm{standard}}\text{–}1)\times 1000\;[\text{‰}]$$

where *R*_sample_ represents the ^13^C:^12^C or ^15^N:^14^N ratio in the sample, and *R*_standard_ represents the ^13^C:^12^C or ^15^N:^14^N ratios of Vienna PeeDee Belemnite or atmospheric N_2_, respectively. Calibration was performed using laboratory standards (CERKU-01, -02, -03) (Tayasu *et al.*, 2011). Analytical standard deviations were <0.09 ‰ for ^13^C (*n* = 24) and <0.22 ‰ for ^15^N (*n* = 24). Total C and N concentrations were determined from sample weights and gas concentrations (CO_2_ and N_2_) based on laboratory standards (Tayasu *et al.*, 2011). To facilitate comparisons with prior studies, enrichment factors (ɛ^13^C and ɛ^15^N) were calculated as the difference between each *C. aphylla* δ^13^C and δ^15^N value and the mean δ^13^C and δ^15^N values of autotrophic reference plants in the same plot (Preiss and Gebauer, 2008).

After confirming no major deviations from normality or homoscedasticity using Q–Q plots and DHARMa simulation-based residual diagnostics, differences in δ^13^C, δ^15^N, ɛ^13^C and ɛ^15^N values among *C. aphylla* coralloid rhizomes, flowers, leaves and autotrophic reference plants were tested using linear mixed models, with plot as a random effect. Pairwise differences were assessed using Tukey–Kramer *post hoc* tests. Each response variable was analysed separately. Statistical analyses were performed in R (R Core Team, 2025). A linear two-source mixing model was used to estimate the proportion of carbon derived from fungi in leafy *C. aphylla* (% Cdf): % Cdf = (ɛ^13^C_PMH_/ɛ^13^C_FMH_) × 100. Here, ɛ^13^C_PMH_ is the ^13^C enrichment factor of leaves and flowers of leafy individuals, and ɛ^13^C_FMH_ is the mean ^13^C enrichment factor of flowers from leafless individuals.

## RESULTS

### Chlorophyll concentration and fluorescence

The total chlorophyll concentrations (Chl *a + b*) of *C. aphylla* leaves were quantified as 237.0 ± 49.4 mg m^−2^ (mean ± s.d.). These values did not differ significantly from those of the co-occurring autotrophic plants *A. japonica* var. *japonica* (293.6 ± 48.9 mg m^−2^; *P* = 0.12) and *O. japonica* (263.1 ± 39.1 mg m^−2^; *P* = 0.60) but were significantly higher than those of *R. argyi* (126.7 ± 25.6 mg m^−2^; *P* < 0.01).

Maximum quantum yields of photosystem II (Fv/Fm) in *C. aphylla* were 0.77 ± 0.02, consistent with the typical range for autotrophic plants (0.7–0.83) (Maxwell and Johnson, 2000; Ritchie, 2006). No significant differences in Fv/Fm values were detected among *A. japonica* var. *japonica* (0.78 ± 0.02), *O. japonica* (0.79 ± 0.02) and *R. argyi* (0.78 ± 0.02; *P* > 0.48 for all comparisons).

### Phylogenomic analysis

**Fig. 3.**
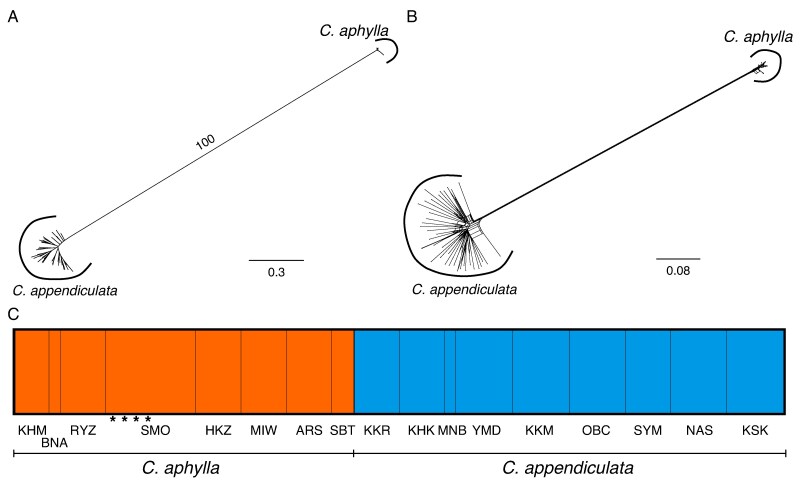
Genomic profiles of *Cremastra aphylla* and its close relative *Cremastra appendiculata* reconstructed from MIG-seq data. (A) Neighbor-Net network based on uncorrected *p*-distances. (B) Maximum-likelihood tree, with intraspecific bootstrap values omitted. (C) Population structure analysis, with species boundaries indicated by thick black vertical lines and population boundaries by thin black lines. Leafy individuals of *C. aphylla* are marked with asterisks in C.

The maximum-likelihood phylogeny based on the full sample set showed that *C. aphylla*, including both leafy and leafless individuals, formed a well-supported clade distinct from *C. appendiculata*, with 100 % bootstrap support. Likewise, the Neighbor-Net network based on the complete dataset revealed two genetically distinct clusters corresponding to the two species (Fig. 3). STRUCTURE analysis of the full sample set at *K* = 2, which yielded the highest Δ*K*, further supported this separation by assigning *C. aphylla* and *C. appendiculata* to distinct genetic groups. These phylogenetic and population structure analyses provided no evidence of genetic admixture between the two species. These findings support the genetic distinctness of *C. aphylla* and *C. appendiculata*, validating their recognition as separate species. This also excludes the possibility that leafy *C. aphylla* individuals arose via hybridization or introgression.

**Fig. 4.**
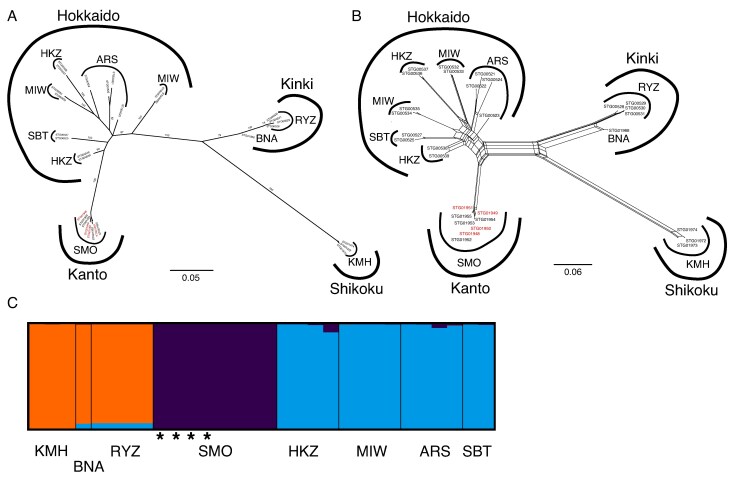
Genomic profiles of *Cremastra aphylla* reconstructed from MIG-seq data. (A) Neighbor-Net network based on uncorrected *p*-distances. (B) Maximum-likelihood tree, with bootstrap values of <70 % omitted. (C) Population structure analysis, with population boundaries indicated by thin black lines. Leafy individuals of *C. aphylla* are marked with red labels in A and B and with asterisks in C. Sample ID information is provided in Supplementary Data Table S1.

In the maximum-likelihood phylogeny and Neighbor-Net network based on the *C. aphylla*-only subset, leafy individuals clustered within a monomorphic clade together with leafless individuals from the Shimoongatamachi population, where both morphs co-occur (Fig. 4). Within this clade, leafy individuals were genetically indistinguishable from their leafless counterparts. These results indicate that, at least within this population, leaf production reflects phenotypic plasticity rather than a genetically divergent lineage. STRUCTURE analysis of the *C. aphylla*-only subset at *K* = 3, which yielded the highest Δ*K*, identified the Shimoongatamachi population as one of three genetic clusters. However, genetic structure across all analyses, including STRUCTURE, primarily reflected geographical proximity among sampling localities (e.g. Hokkaido, Kanto, Kinki and Shikoku). Moreover, the maximum-likelihood phylogeny and Neighbor-Net network based on both the full dataset and the *C. aphylla*-only subset revealed that all individuals from Shimoongatamachi were nested within clades composed exclusively of leafless individuals from other regions. Although it cannot be entirely ruled out that the frequent leaf production in the Shimoongatamachi population might reflect genetic changes, occasional leaf development has also been observed in *C. aphylla* individuals from other regions (not included in this study). Taken together, these findings suggest that leafy individuals do not represent a distinct taxon.

### Molecular identification of mycorrhizal fungi

**Fig. 5.**
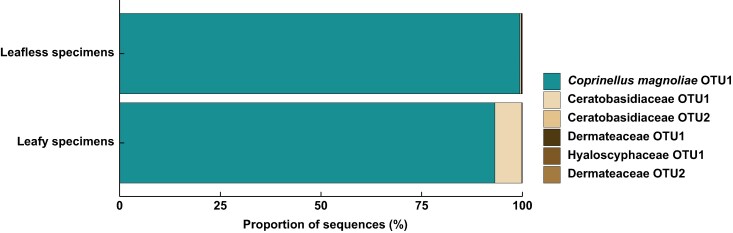
Relative abundance of mycorrhizal communities associated with leafless and leafy specimens of *Cremastra aphylla*.

Metabarcoding analysis revealed that both leafless and leafy *C. aphylla* predominantly associate with a fungus from the family Psathyrellaceae (Fig. 5 and Supplementary Data Table S2). After quality filtering, four OTUs were identified from the mycorrhizal tissues of leafless individuals, comprising 86 088 sequencing reads. Among these, a single OTU assigned to *Coprinellus* (Psathyrellaceae) accounted for 85 552 reads (99.38 %). Likewise, four OTUs (29 852 reads) were identified in leafy individuals, with a *Coprinellus* OTU comprising 27 814 reads (93.17 %). In addition, two OTUs belonging to Ceratobasidiaceae were detected in leafy individuals (2023 reads; 6.78 %) but were rare in leafless individuals (238 reads; 0.27 %). The remaining OTUs, represented by a few reads and limited occurrences, are likely to correspond to opportunistic fungi of negligible ecological relevance.

**Fig. 6.**
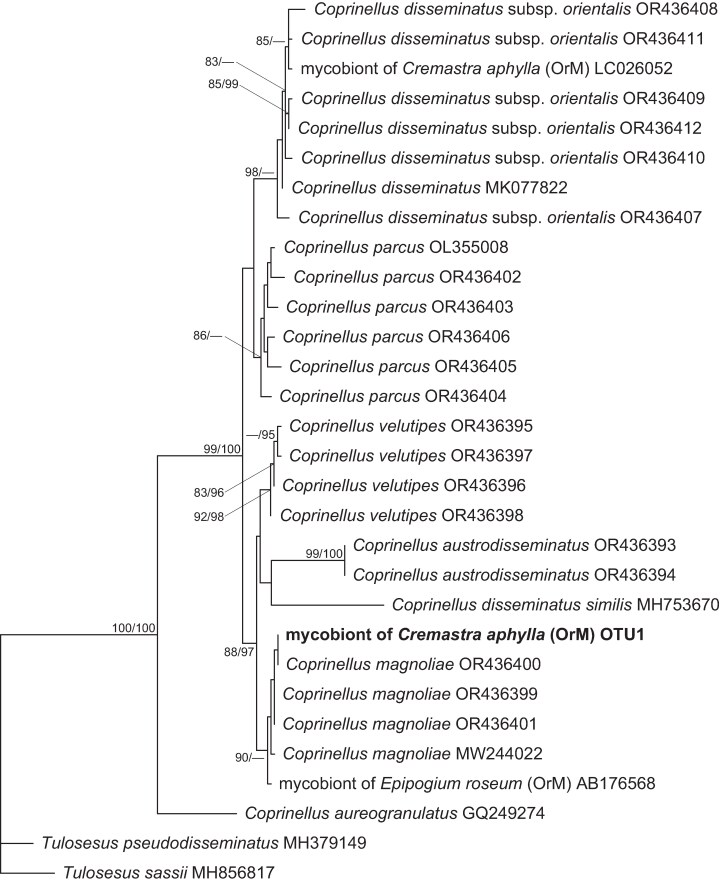
Phylogenetic tree of ITS2 rDNA sequences from the *Coprinellus* operational taxonomic unit detected in the mycorrhizal samples of *Cremastra aphylla* (highlighted in bold), alongside sequences retrieved from the INSDC database. Accession numbers are provided for all INSDC sequences. The tree is rooted using *Tulosesus pseudodisseminatus* and *Tulosesus sassii* (Psathyrellaceae) as outgroups. Nodes with SH-aLRT support values of <80 % and ultrafast bootstrap values of <95 % are not displayed. The scale bar indicates the number of substitutions per site. Abbreviation: OrM, Orchid mycorrhizal fungi.

Molecular phylogenetic analysis revealed that the dominant *Coprinellus* OTU in *C. aphylla* formed a clade with *Coprinellus magnoliae*, a member of the *C. disseminatus* species complex. This clade also included a fungal symbiont of the unrelated fully mycoheterotrophic orchid *Epipogium roseum* (Fig. 6).

### Stable isotope analysis

**Fig. 7.**
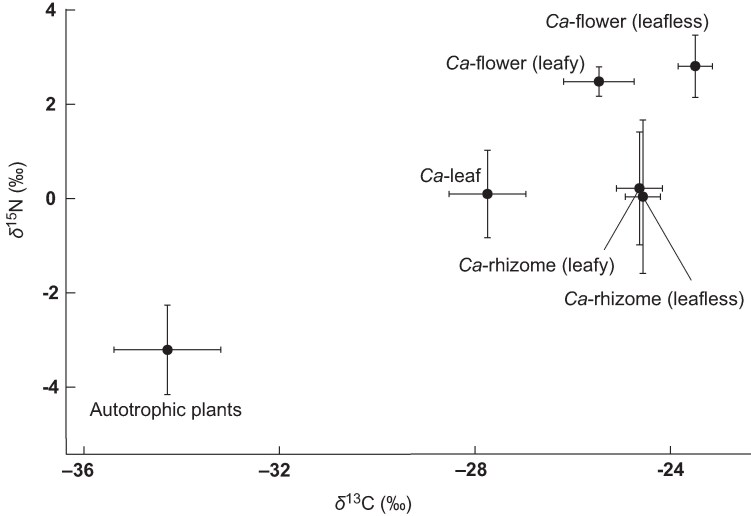
Mean (±s.d.) values of δ^13^C and δ^15^N in *Cremastra aphylla* and its neighbouring autotrophic plants. Abbreviations: *Ca*-flower (leafy/leafless), flowers of leafy or leafless *C. aphylla* individuals; *Ca*-leaf, leaves of leafy *C. aphylla* individuals; *Ca*-rhizome (leafy/leafless), coralloid rhizomes of leafy or leafless *C. aphylla* individuals.

All *C. aphylla* specimens (−23.5 ± 0.4 ‰ in flower specimens of leafless individuals, *n* = 10; −25.5 ± 0.7 ‰ in flower specimens of leafy individuals, *n* = 5; −24.6 ± 0.4 ‰ in coralloid rhizome specimens of leafless individuals, *n* = 5; −24.6 ± 0.5 ‰ in coralloid rhizome specimens of leafy individuals, *n* = 3; and −27.7 ± 0.8 ‰ in leaf specimens of leafy individuals, *n* = 6) displayed significantly higher δ^13^C values than autotrophic reference plants (−34.3 ± 1.1 ‰, *P* < 0.001 for all comparisons; Supplementary Data Table S3). Flowers of leafless individuals showed significantly higher δ^13^C values than both flowers and leaves of leafy individuals (*P* < 0.001 for both). Flowers of leafy individuals also had higher δ^13^C values than their leaves (*P* < 0.001; Fig. 7). No significant δ^13^C differences were observed between rhizomes of leafless and leafy individuals (*P* = 1.00). Using the mean ɛ^13^C of leafless flowers as the reference for full mycoheterotrophy, photosynthesis was estimated to contribute 38.0 ± 8.1 % of carbon in the leaves and 17.7 ± 9.0 % in the flowers of leafy individuals.

Likewise, all *C. aphylla* specimens exhibited significantly higher δ^15^N values (2.8 ± 0.7 ‰ in flower specimens of leafless individuals, 2.5 ± 0.3 ‰ in flower specimens of leafy individuals, 0.0 ± 1.6 ‰ in coralloid rhizome specimens of leafless individuals, 0.2 ± 1.2 ‰ in coralloid rhizome specimens of leafy individuals, and 0.1 ± 0.9 ‰ in leaf specimens of leafy individuals) than autotrophic reference plants (−3.2 ± 0.9 ‰, *P* <0.001 for all comparisons; Supplementary Data Table S3). Flowers of both leafless and leafy individuals had significantly higher δ^15^N values than the leaves of leafy individuals (*P* < 0.001 for both) but did not differ from each other (*P* = 0.99). Rhizomes from leafless and leafy individuals also showed no significant differences (*P* = 1.00).

## DISCUSSION

Our study provides insights into the physiological and evolutionary ecology of leafy *C. aphylla* by integrating phylogenomics, stable isotope analysis, chlorophyll measurements and fungal metabarcoding. Phylogenomic analyses showed no genomic admixture with leafy *C. appendiculata*, ruling out hybridization as the origin of leafy *C. aphylla*. Additionally, leafy individuals were genetically indistinguishable from their leafless counterparts within the same population. This suggests that, at least in the studied population, leaf development in *C. aphylla* reflects phenotypic plasticity, although the environmental or physiological factors underlying this trait remain unclear. Furthermore, given that genetic structure corresponded to geographical proximity and that no other morphological differences were evident, leafy *C. aphylla* is unlikely to represent a taxonomically distinct entity.

Stable isotope analysis revealed marked ^13^C enrichment in both leafless and leafy *C. aphylla* individuals relative to neighbouring autotrophic plants. The ^13^C enrichment values [10.9 ± 0.6 ‰ in flowers of leafless individuals (*n* = 10), 9.0 ± 1.0 ‰ in flowers of leafy individuals (*n* = 5) and 6.8 ± 0.9 ‰ in leaves of leafy individuals (*n* = 6)] fall within the ranges reported for fully mycoheterotrophic orchids associated with ectomycorrhizal, litter-decaying and wood-decomposing fungi (7.8 ± 1.6, 8.2 ± 0.5 and 11.0 ± 2.3 ‰, respectively) and for protocorms of the related *C. appendiculata* exploiting *Coprinellus* fungi (8.0 ± 0.6 ‰) (Hynson *et al.*, 2016; Zahn *et al.*, 2022). However, the lower enrichment observed in leafy individuals reflects the signal of ^13^C-depleted photosynthates, indicating a contribution from photosynthesis.

This contrasts somewhat with the leafy forms of the typically leafless *Pyrola aphylla*, which exhibit isotopic signatures indicative of full or near-full mycoheterotrophy (Hynson *et al.*, 2009). Using the mean ɛ^13^C values of flowers from leafless *C. aphylla* as the fully mycoheterotrophic end point, the proportion of carbon derived from photosynthesis was estimated to be ∼40 % in the leaves of leafy individuals. Chlorophyll measurements confirmed that these leaves accumulate chlorophyll at levels similar to those of co-occurring green understorey species and *C. appendiculata*. Chlorophyll fluorescence analyses further demonstrated no reduction in photosystem II efficiency relative to other photosynthetic plants, including *C. appendiculata* (Maxwell and Johnson, 2000; Suetsugu and Okada, 2025*b*). Although the fungal carbon contribution in leafy *C. aphylla* (∼60 %) exceeds that of *C. appendiculata* (∼50 %), this is likely to reflect reduced leaf biomass in *C. aphylla* (∼10 cm in length) compared with *C. appendiculata* (∼30 cm in length), rather than a decrease in photosynthetic capacity (Yagame *et al*., 2021; Suetsugu and Okada, 2025*b*).

In fully mycoheterotrophic plants, leaves are typically reduced to achlorophyllous scales (Tsukaya, 2018), yet *C. aphylla* and *P. aphylla* occasionally produce functional leaves (Hynson *et al.*, 2009), suggesting that full mycoheterotrophy might evolve before complete loss of photosynthetic function. Notably, the plastome of *C. aphylla* has lost only *ndh* genes (Lu *et al.*, 2025). Although *ndh* loss often marks the early stage of plastome degradation in heterotrophs, it also occurs in shade-adapted autotrophs (Goedderz *et al.*, 2024). Further reductions involving photosynthetic (*psa*, *psb*, *pet*, *rbcL* and *rpo*), ATP synthase and housekeeping genes (*matK* and *rpl*) are common in fully heterotrophic plants (Graham *et al.*, 2017; Barrett *et al.*, 2019). However, loss limited to *ndh* genes is also found in other leafless but photosynthetically competent orchids, such as *Cymbidium macrorhizon* and *Limodorum abortivum* (Kim *et al.*, 2020; Lu *et al.*, 2025). These observations support the hypothesis that leaf reduction might precede the loss of photosynthetic capacity. The occurrence of maladaptive albino individuals in leafy, partially mycoheterotrophic orchids also suggests that loss of photosynthesis without leaf reduction might reduce fitness (Roy *et al.*, 2013).

We estimated that flowers of leafy *C. aphylla* contain ∼20 % carbon derived from photosynthesis, suggesting translocation of photosynthates from leaves to flowers. In contrast, no δ^13^C differences were observed in coralloid rhizomes between leafless and leafy individuals, indicating that these underground organs are entirely sustained by fungal carbon, regardless of leaf presence. This supports the idea that, in partially mycoheterotrophic orchids, photosynthates are preferentially allocated to reproductive organs rather than to subterranean structures (Gonneau *et al.*, 2014; Suetsugu *et al.*, 2018; Lallemand *et al.*, 2019*a*). Even in partially mycoheterotrophic genera such as *Cephalanthera*, *Epipactis* and *Gentiana*, which allocate more resources to leaf development than *C. aphylla*, the photosynthetic contribution to subterranean tissues remains limited (Gonneau *et al.*, 2014; Lallemand *et al.*, 2019*a*; Suetsugu, 2025). The anatomical or physiological mechanisms driving this pattern remain an important topic for future investigation.

Fungal metabarcoding data confirmed that both leafless and leafy individuals are predominantly colonized by a single Psathyrellaceae OTU. Although mycorrhizal associations are often diffuse (Smith and Read, 2008), high fungal specificity is typical of fully mycoheterotrophic plants. This specificity might result from plant-mediated partner selection or fungal exclusion of parasitic interactions (Bruns *et al.*, 2002; Egger and Hibbett, 2004; Hynson *et al.*, 2009). The high specificity observed in leafy *C. aphylla* and in other leafless orchids retaining residual photosynthesis suggests that fungal specialization can precede the evolution of full mycoheterotrophy (Girlanda *et al.*, 2006; Zimmer *et al.*, 2008; Suetsugu *et al.*, 2018). Nonetheless, the detection of minor reads from Ceratobasidiaceae, putatively orchid mycorrhizal fungi, in leafy individuals might suggest a partial relaxation of fungal specificity in these forms.

The closely related *C. appendiculata* also exhibits trophic plasticity, influenced by chlorophyll content, the presence of coralloid rhizomes and fungal partners (Yagame *et al.*, 2021; Zahn *et al.*, 2022; Suetsugu *et al.*, 2022; Suetsugu and Okada, 2025*b*). Green and variegated individuals with coralloid rhizomes show a high level of mycoheterotrophy, deriving more than half of their carbon from Psathyrellaceae fungi (Yagame *et al.*, 2021; Suetsugu *et al.*, 2022; Suetsugu and Okada, 2025*b*). In contrast, variegated individuals lacking coralloid rhizomes are largely autotrophic and associate predominantly with rhizoctonias, underscoring the role of coralloid rhizomes in facilitating mycoheterotrophy (Suetsugu and Okada, 2025*b*).

These findings suggest that both species maintain a flexible balance between autotrophy and mycoheterotrophy. Although the genetic mechanisms underlying this flexibility in *C. appendiculata* remain unclear, it might represent an evolutionary precursor to the enhanced mycoheterotrophy in *C. aphylla*. However, increased autotrophy in *C. appendiculata* is linked to the absence of both coralloid rhizomes and Psathyrellaceae associations (Yagame *et al.*, 2021; Zahn *et al.*, 2022; Suetsugu *et al.*, 2022; Suetsugu and Okada, 2025*b*), whereas *C. aphylla* consistently retains both traits regardless of leaf presence. This suggests that its small leaves alone are insufficient to support predominantly autotrophic growth.

### Conclusion

In conclusion, our integrated findings support partial mycoheterotrophy in leafy individuals of *C. aphylla*. Phylogenetic analyses confirmed that leafy individuals bearing much smaller leaves (∼10 cm in length) than those of *C. appendiculata* can be identified reliably as *C. aphylla* based on key traits such as dark purple flowers, a rugose–verrucose callus on the labellum and the absence of ventral wings on the column. Isotopic and chlorophyll fluorescence data suggest that leaf production can shift *C. aphylla* from full to partial mycoheterotrophy, although leafy individuals still depend heavily on fungal carbon. The contrasting traits and trophic plasticity of *C. aphylla* and *C. appendiculata* make them useful models for studying the evolution of mycoheterotrophy and leaf reduction. Their saprotrophic associations also make them suitable for *in vitro* cultivation (e.g. Yagame *et al.*, 2024), offering a promising system for exploring physiological transitions along the mycoheterotrophic continuum.

## Supplementary Material

mcaf235_Supplementary_Data

## Data Availability

MIG-seq and fungal community data have been deposited in the Sequence Read Archive under accession numbers PRJDB35934 and PRJNA1213263, respectively.
